# Jailed Balloon Technique Is Superior to Jailed Wire Technique in Reducing the Rate of Side Branch Occlusion: Subgroup Analysis of the Conventional Versus Intentional StraTegy in Patients With High Risk PrEdiction of Side Branch OccLusion in Coronary Bifurcation InterVEntion Trial

**DOI:** 10.3389/fcvm.2022.814873

**Published:** 2022-03-31

**Authors:** Dong Zhang, Zhiyong Zhao, Guofeng Gao, Han Xu, Hao Wang, Shuai Liu, Dong Yin, Lei Feng, Chenggang Zhu, Yang Wang, Yanyan Zhao, Yuejin Yang, Runlin Gao, Bo Xu, Kefei Dou

**Affiliations:** ^1^State Key Laboratory of Cardiovascular Disease, Beijing, China; ^2^Department of Cardiology, National Center for Cardiovascular Diseases, Fu Wai Hospital, Beijing, China; ^3^Chinese Academy of Medical Sciences and Peking Union Medical College, Beijing, China; ^4^Medical Research and Biometrics Center, National Center for Cardiovascular Diseases, Beijing, China; ^5^Catheterization Laboratories, Fu Wai Hospital, Beijing, China

**Keywords:** coronary bifurcation lesions, jailed balloon technique, jailed wire technique, side branch occlusion, major adverse cardiac event (MACE)

## Abstract

**Objective:**

Jailed balloon technique (JBT) is an active side branch (SB) protection strategy and is considered to be superior to the jailed wire technique (JWT) in reducing SB occlusion. However, no randomized trials have proved that. We aim to investigate whether JBT could decrease the SB occlusion rate.

**Methods:**

Conventional versus Intentional straTegy in patients with high Risk prEdiction of Side branch OccLusion in coronary bifurcation interVEntion (CIT-RESOLVE) (NCT02644434, registered on December 31, 2015) (https://clinicaltrials.gov) is a randomized trial that assessed the effects of different strategies on SB occlusion rate in patients with a high risk of SB occlusion. The present subgroup analysis enrolled bifurcation lesions (2 mm ≤ reference vessel diameter of SB < 2.5 mm) with Visual estimation for Risk prEdiction of Side branch OccLusion in coronary bifurcation intervention (V-RESOLVE) score ≥ 12 points. The primary endpoint is SB occlusion. One-year clinical events were compared.

**Results:**

A total of 284 subjects at 16 sites were randomly assigned to the JBT group (*n* = 143) or the JWT group (*n* = 141). The rate of SB occlusion (9.1 vs. 19.9%, *p* = 0.02) and periprocedural myocardial infarction (defined by WHO, 7 vs. 14.9%, *p* = 0.03) is significantly lower in the JBT group than in the JWT group. The JBT and JWT groups showed no significant differences in cardiac death (0.7 vs. 0.7%, *p* = 1), myocardial infarction (MI, 6.3 vs. 7.1%, *p* = 0.79), target lesion revascularization (TLR, 1.4 vs. 2.1%, *p* = 0.68), and major cardiac adverse events (MACE, a composite of all-cause death, MI, or TLR, 8.4 vs. 10.6%, *p* = 0.52) during a 1-year follow-up.

**Conclusion:**

In patients with a high risk of SB occlusion (V-RESOLVE score ≥ 12 points), JBT is superior to JWT in reducing SB occlusion. However, no significant differences were detected in 1-year MACE.

## Introduction

Side branch (SB) occlusion may be a disaster during coronary bifurcation intervention and could lead to serious adverse clinical events (1, 2). Protecting the SB to keep it open is one of the main principles when performing bifurcation lesion intervention (3, 4). Dedicated bifurcation techniques, such as jailed wire technique (JWT), jailed balloon technique (JBT), and jailed corsair technique have been proposed to help SB protection (5). Among them, JWT is widely used. Nevertheless, JBT is considered to be more effective in the preservation of SB patency. Although the JWT has been used in numerous bifurcations and the JBT has been proposed for about 10 years (6), to the best of our knowledge, no randomized trials have been performed to compare the rate of SB occlusion between JBT and JWT during a high-risk coronary bifurcation intervention.

The present study aims to investigate whether JBT could decrease the rate of SB occlusion and the following adverse events by performing a subgroup analysis of the CIT-RESOLVE Trial.

## Materials and Methods

### Design

CIT-RESOLVE (https://clinicaltrials.gov; NCT02644434) was an investigator-initiated, prospective, multicenter, single-blinded (patients were masked), randomized controlled trial conducted at 16 hospitals in China. Details of the design, population, outcome definitions, and 1-month clinical outcomes have been published previously (7, 8).

A total of 335 patients who had high-risk coronary bifurcation lesion (V-RESOLVE score ≥ 12 points) requiring stent implantation were stratified by reference vessel diameter (RVD) of the SB and randomly assigned to the active strategy group or conventional strategy group in a 1:1 ratio. The choice of devices and the utilization of intravascular imaging were at the physician’s discretion. Periprocedural antiplatelet and antithrombotic medications were administered according to current guidelines. Patients in the conventional strategy group were treated using either JWT (2 mm ≤ SB RVD < 2.5 mm) or a provisional two-stent strategy (SB RVD ≥ 2.5 mm), while patients in the active strategy group were treated by either JBT (2 mm ≤ SB RVD < 2.5 mm) or elective two-stent strategy (SB RVD ≥ 2.5 mm).

### Patients

This analysis addresses the subgroup of high-risk patients with RVD ≥ 2 mm and < 2.5 mm and underwent JBT (assigned to the active strategy group) or JWT (assigned to the conventional strategy group).

The study complied with the Declaration of Helsinki. The study protocol was approved by the investigational review board or ethics committee at each site. All patients provided written informed consent.

### Interventional Procedures

All procedures were performed by experienced interventionalists. Coronary angioplasty and treatment therapy were done according to standard techniques, and the choice of devices and the utilization of intravascular imaging were at the physician’s discretion. Periprocedural antiplatelet and antithrombotic medications were administered according to current guidelines. After the percutaneous coronary intervention (PCI) procedure, patients were prescribed 100 mg of aspirin daily indefinitely and 75 mg of clopidogrel daily for at least 12 months.

#### Jailed Balloon Technique

JBT was performed according to previously described details (6, 9). Vessel wiring for both main vessel (MV) and SB and lesion preparation were performed as necessary. When a crossover SB stent was placed in the MV, a balloon with appropriate size was introduced into the ostial SB vessel. The MV stent and SB balloon positions were adjusted to ensure a 2-mm distance between the balloon proximal marker and the MV stent, and the MV stent was then deployed. Coronary angiography was used to assess SB blood flow; if there is thrombolysis in myocardial infarction (TIMI) flow grade decrease in SB, then the balloon in proximal SB was inflated with low pressure (< 3 atm). The jailed wire and balloon were retracted after successful rewiring into the SB. The proximal optimization technique (POT) was recommended to achieve a good apposition of the proximal MV stent. A kissing balloon was performed at the interventionalist’s discretion regardless of the absence/presence of SB compromise. A T and protrusion stenting followed by kissing balloon dilatation was recommended if SB stenting was deemed necessary.

#### Jailed Wire Technique

Coronary guide wires were inserted into the MV and SB, and lesion preparation was then at the operator’s discretion. The MV stent was located and released with wire protection in SB. After MV stenting, coronary angiography was performed to ascertain if SB needed further treatment in case of SB occlusion, TIMI flow decrease, or SB dissection greater than type A. If post-processing of the SB was required, the wire was replaced through the stent mesh into the SB, followed by balloon dilation and final kissing balloon or further SB stenting to restore SB blood flow.

### Events

The primary endpoint, SB occlusion, was defined as any decrease in the TIMI flow grade or an absence of flow in the SB immediately after full apposition of the MV stent to the vessel wall (1, 2). Secondary endpoints included rate of periprocedural MI, as defined by Society for Cardiovascular Angiography and Interventions (SCAI) (10), WHO, (11) and Academic Research Consortium-2 (ARC-2) (12) criteria and major adverse cardiac events (MACE), a composite of all-cause death, myocardial infarction (MI), or target vessel revascularization at each follow-up time point. All adverse clinical events were adjudicated by an independent clinical events committee.

### Statistics

All statistical analyses were performed in both intention-to-treat (ITT) population and as-treated set (ATS). Continuous variables are presented as mean ± *SD* and categorical variables as counts and percentages. Group differences were analyzed using Student’s *t*-test for normally distributed continuous variables and by the Chi-square or Fisher exact tests for categorical variables. The 95% CIs of the differences between the two treatment arms were calculated by using normal approximation for continuous variables and the Wald asymptotic method for binary variables. For primary endpoint analysis, 95% CIs of the difference between the two treatment arms were calculated by the Cochran-Mantel-Haenszel Chi-square test with adjustment for central effects. Rate-free survival from MACE was calculated by the Kaplan-Meier analysis and was compared using the log-rank test.

All the statistical analyses were performed using SAS software, version 9.4 (SAS Institute, Cary, North Carolina, United States). A two-sided *p*-value of 0.05 was considered significant.

## Results

### Patients

**FIGURE 1:**
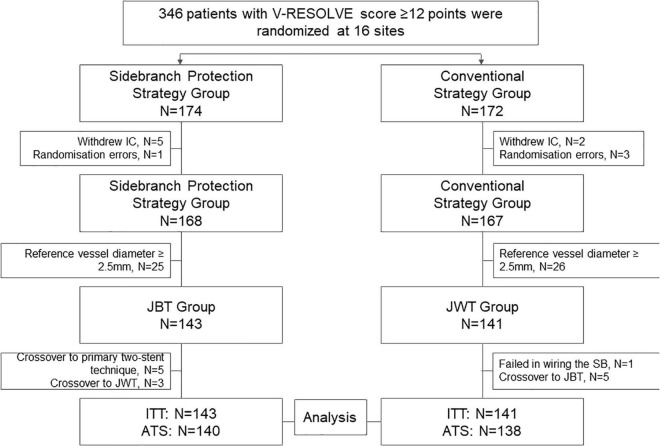
Study population. From December 2016 to April 2019, a total of 335 subjects were randomly assigned to the active strategy group (*n* = 168) or conventional strategy group (*n* = 167). Among them, 143 patients in the active strategy group have an SB with 2 mm ≤ RVD < 2.5 mm and were assigned to JBT accordingly, while 141 patients in the conventional strategy group have an SB with 2 mm ≤ RVD < 2.5 mm and were assigned to JWT. For the ATS, 140 patients underwent the JBT strategy and 138 patients underwent the JWT strategy. IC, informed content; JBT, jailed balloon technique; JWT, jailed wire technique; ITT, intention to treat; ATS, as treated set.

From December 2016 to April 2019, a total of 335 subjects were randomly assigned to the active strategy group (*n* = 168) or to the conventional strategy group (*n* = 167). The follow-up of the last patient ended in May 2020. Among them, 143 patients in the active strategy group have an SB with 2 mm ≤ RVD < 2.5 mm and were assigned to JBT accordingly, while 141 patients in the conventional strategy group have an SB with 2 mm ≤ RVD < 2.5 mm and were assigned to JWT. In the JBT subgroup, five subjects crossed over to a primary two-stent technique and 3 subjects crossed over to JWT. In the JWT subgroup, one subject failed in wiring the SB, and 5 subjects crossed over to JBT because operators realized the high risk of SB occlusion and insisted on an active strategy. Thus, for the ATS, 140 patients underwent the JBT strategy and 138 patients underwent the JWT strategy (Figure 1).

Demographic characteristics are shown in Table 1. The mean age of the patients was 61 years, and 16.4% (55/335) had a history of previous MI. Patients in the JBT group had more unstable angina than those in the JWT group (67.1 vs. 53.9%, *p* = 0.02). Other baseline characteristics were balanced between the two treatment groups.

**TABLE 1:** Baseline characteristics (intention-to-treat population).

	JBT group (*N* = 143)	JWT group (*N* = 141)	*P*-value
Age, years	61.1 ± 9.1	60.9 ± 10.0	0.91
Male	74.8% (107)	68.1% (96)	0.21
Body mass index	27.5 ± 25.6 (138*)	25.4 ± 6.0 (137*)	0.38
Diabetes mellitus	28.0% (40)	29.8% (42)	0.74
Hypertension	56.6% (81)	66.0% (93)	0.11
Hyperlipidemia	39.2% (56)	40.4% (57)	0.83
Current smoker	46.2% (66)	42.6% (60)	0.54
Previous MI	22.4% (32)	23.4% (33)	0.84
Previous PCI	14.0% (20)	18.4% (26)	0.31
Previous CABG	0.7% (1)	0.7% (1)	1.0
Family history of CAD	9.1% (13)	12.8% (18)	0.32
Peripheral arterial disease	8.4% (12)	7.8% (11)	0.86
Unstable angina	67.1% (96)	53.9% (76)	0.02
Left ventricular ejection fraction	63.2 ± 8.6 (136*)	61.3 ± 9.3 (126*)	0.10

Values are mean ± SD or% (n). *Number of patients for whom continuous variables were calculated. JBT, jailed balloon technique; JWT, jailed wire technique; MI, myocardial infarction; PCI, percutaneous coronary intervention; CABG, coronary artery bypass grafting; CAD, coronary artery disease.

### Lesion and Procedural Characteristics

Lesion characteristics are shown in Table 2. Most of the target lesions were located in the anterior descending branch and were true bifurcation lesions (Medina 1,1,1, or 1,0,1, or 0,1,1). The baseline SYNTAX (Synergy between Percutaneous Coronary Intervention with Taxus and Cardiac Surgery) score was 17.5 ± 7.6 in the JBT group and 18.3 ± 7.8 in the JWT group (*p* = 0.42). There is more incidence of irregular plaque in the JBT group. No other significant differences were detected regarding lesion characteristics.

**TABLE 2:** Lesion characteristics (intention-to-treat population).

	JBT group (*N* = 143)	JWT group (*N* = 141)	*P*-value
Multivessel disease	52.4% (75)	52.5% (74)	1.0
**Target lesion location**			
Left anterior descending/diagonal	85.3% (122)	83.0% (117)	0.59
Left circumflex/obtuse marginal	12.6% (18)	13.5% (19)	0.82
Right coronary artery bifurcation	2.1% (3)	3.5% (5)	0.50
**Medina classification**			
1,0,0	2.1% (3)	2.1% (3)	1.0
0,1,0	1.4% (2)	1.4% (2)	1.0
1,1,0	7.7% (11)	4.3% (6)	0.22
1,1,1	62.2% (89)	63.1% (89)	0.88
0,0,1	0% (0)	0% (0)	-
1,0,1	10.5% (15)	11.3% (16)	0.82
0,1,1	16.1% (23)	17.7% (25)	0.71
ACC/AHA B2/C lesions	95.1% (136)	96.5% (136)	0.57
Baseline SYNTAX score	17.5 ± 7.6 (141*)	18.3 ± 7.8 (138*)	0.42
**MV qualitative analysis**			
Baseline TIMI flow			0.29
0	4.2% (6)	5.0% (7)	
I	4.2% (6)	7.8% (11)	
II	4.2% (6)	7.8% (11)	
III	87.4% (125)	79.4% (112)	
In-stent restenosis	0% (0)	2.1% (3)	0.12
Total occlusion	3.5% (5)	5.0% (7)	0.54
Moderate or heavy calcification	7.7% (11)	9.2% (13)	0.64
Severely tortuous or angulated lesion	21.7% (31)	20.6% (29)	0.82
Thrombus containing	1.4% (2)	0 (0)	0.50
Plaque located at the same side of SB	93.0% (133)	90.8% (128)	0.49
Irregular plaque	57.3% (82)	59.6% (84)	0.70
**SB qualitative analysis**			
Baseline TIMI flow			0.90
0	0% (0)	0.7% (1)	
I	3.5% (5)	2.8% (4)	
II	5.6% (8)	6.4% (9)	
III	90.9% (130)	90.1% (127)	
In-stent restenosis	0% (0)	0% (0)	-
Total occlusion	0% (0)	0.7% (1)	0.50
Moderate or heavy calcification	0 (0)	2.1% (3)	0.12
Severely tortuous or angulated lesion	13.3% (19)	12.8% (18)	0.90
Thrombus containing	0% (0)	0% (0)	-
Irregular plaque	43.4% (62)	27.0% (38)	0.004
V-RESOLVE score (site)	15.5 ± 3.1 (143*)	15.7 ± 3.2 (141*)	0.65
V-RESOLVE score (core lab)	17.1 ± 3.4 (143*)	17.2 ± 4.3 (141*)	0.80

Values are mean ± SD or% (n). *Number of patients for whom continuous variables were calculated. All the angiographic characteristics were evaluated by the core lab. JBT, jailed balloon technique; JWT, jailed wire technique; ACC, American College of Cardiology; AHA, American Heart Association; SYNTAX, Synergy Between PCI With TAXUS and Cardiac Surgery; MV, main vessel; SB, side branch; TIMI, Thrombolysis in Myocardial Infarction; V-RESOLVE, Visual estimation for Risk prEdiction of Side branch OccLusion in coronary bifurcation intervention.

Procedural characteristics are shown in Table 3. The majority of the procedures were performed by the transradial approach. All stents used in the present study are second-generation drug-eluting stents with an open-cell design. The procedural details in the MV were similar between the two groups. Regarding the SB, JBT was applied in most of the patients (94.4%) in the JBT group, while only 3.5% (5/141) of the patients in the JWT group crossed over to the JBT technique. Similar rates of lesion success were found in the two groups. The results of ATS and quantitative coronary angiography (QCA) are shown in Supplementary Tables 1–3.

**TABLE 3:** Procedural characteristics and results (intention-to-treat population).

	JBT group (*N* = 143)	JWT group (*N* = 141)	Difference (95% CI)*	*P*-value
Transradial approach	97.2% (139)	95.0% (134)	2.2 (–2.3, 6.7)	0.34
Nitroglycerin use	32.9% (47)	27.7% (39)	5.2 (–5.5, 15.9)	0.34
Dopamine use	0.7% (1)	0% (0)	0.7 (–0.7, 2.1)	1.00
**MV**				
Balloon pre-dilation	100% (143)	98.6% (139)	1.4 (–0.5, 3.4)	0.25
Maximal diameter of pre-dilation balloon, mm	2.3 ± 1.1	2.4 ± 1.6	–0.1 (–0.4, 0.2)	0.48
Maximal inflation pressure with pre-dilation balloon, atm	12.1 ± 2.5	11.9 ± 2.8	0.3 (–0.4, 0.9)	0.38
Dissection before MV stenting	2.1% (3)	4.3% (6)	–2.2 (–6.2, 1.9)	0.33
Number of stents in MV	1.3 ± 0.5	1.3 ± 0.5	0.1 (–0.04, 0.2)	0.56
Stent diameter in MV, mm	3.0 ± 0.4	3.0 ± 0.3	0.07 (–0.01, 0.15)	0.09
Stent diameter/distal main vessel diameter	1.31 ± 0.29	1.36 ± 0.29	–0.05 (–0.13, 0.02)	0.13
Total stent length in MV, mm	26.8 ± 7.8	27.5 ± 8.8	–0.7 (–2.7, 1.2)	0.46
Lesion success	99.3% (142)	99.3% (140)	–0.01 (–1.94, 1.96)	1.00
**SB**				
Balloon pre-dilation	39.2% (56)	34.0% (48)	5.1 (–6.1, 16.3)	0.37
SB stenting				
Elective 2-stent strategy	3.5% (5)	0 (0)	3.5 (0.5, 6.5)	0.06
Provisional SB stenting	0.7% (1)	2.1% (3)	–1.4 (–4.2, 1.3)	0.37
Number of stents in SB	0.03 ± 0.18	0.04 ± 0.22	0 (–0.05, 0.05)	0.98
Jailed balloon technique	94.4% (135)	3.5% (5)	90.9 (86.0, 95.7)	< 0.001
Jailed balloon diameter, mm	1.82 ± 0.24	1.80 ± 0.27	0.02 (–0.2, 0.24)	
Jailed balloon length, mm	14.95 ± 0.61	15.00 ± 0.00	–0.05 (–0.6, 0.49)	
Jailed balloon required inflation	16.8% (24)	1.4% (2)	15.36 (8.94,21.79)	< 0.001
Treatment after MV stent deployed	45.5% (65)	34.8% (49)	10.7 (–0.7, 22.0)	0.07
Final kissing balloon inflation	20.3% (29)	18.4% (26)	1.8 (–7.4, 11.0)	0.69
Lesion treated with POT	32.1% (45)	30.0% (42)	2.1 (–8.7, 13.0)	0.70
Lesion treated with re-POT	3.6% (5)	1.4% (2)	2.1 (–1.5, 5.8)	0.45
Lesion success	93.7% (134)	92.9% (131)	0.8 (–5.0, 6.6)	0.79

Values are mean ± SD or% (n). *The value is the difference between the intentional strategy group and the conventional strategy group. CI, confidence interval; RVD, reference vessel diameter; POT, proximal optimization technique; other abbreviations as in Table 2.

### Primary Endpoints

**FIGURE 2:**
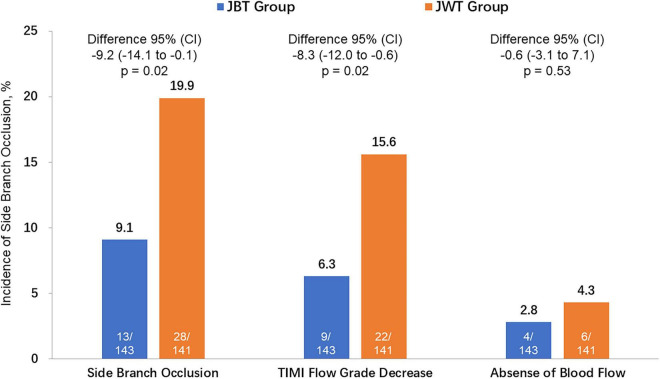
The incidence of the primary endpoint [side branch (SB) occlusion] and its 2 components [TIMI (thrombolysis in myocardial infarction) flow grade decrease and absence of blood flow] between the SB protection jailed balloon technique (JBT) group and jailed wire technique (JWT) group. CI, confidence interval.

By ITT analysis, the primary endpoint of SB occlusion occurred in 13 patients (9.1%) in the JBT group and in 28 patients (19.9%) in the JWT group (risk difference –9.2% [95% CI: –14.1 to –0.1%], *p* = 0.02; odds ratio 0.40 [95% CI: 0.20–0.82]) (Table 4 and Figure 2). The difference was mainly driven by a significantly lower TIMI flow grade decrease rate in the JBT strategy group (6.3 vs. 15.6%, *p* = 0.02). Similar results were found in the ATS population (Table 4).

**TABLE 4:** Primary endpoint (intention-to-treat and as-treated populations).

	JBT group	JWT group	Difference (95% CI)*	*P*-value
**Intention-to-treat population**				
Overall population	*N* = 143	*N* = 141		
Side branch occlusion	9.1% (13)	19.9% (28)	–9.2% (–14.1%, –0.1%)	0.02
TIMI flow grade decrease	6.3% (9)	15.6% (22)	–8.3% (–12.0%, –0.6%)	0.02
Absence of blood flow	2.8% (4)	4.3% (6)	–0.6% (–3.1%, 7.1%)	0.53
**As-treated set**				
Overall population	*N* = 140	*N* = 138		
Side branch occlusion	8.6% (12)	21.0% (29)	–12.0% (–16.0%, –4.7%)	0.004
TIMI flow grade decrease	6.4% (9)	15.9% (22)	–6.8% (–11.1%, –1.3%)	0.01
Absence of blood flow	2.1% (3)	5.1% (7)	–1.8% (–4.0%, 5.0%)	0.20

Values are% (n). The Student’s t-test with center adjustment was used for comparison between groups. *The value is the difference between intentional strategy group and conventional strategy group. Abbreviations as in Table 2.

### Periprocedural Myocardial Infarction

Periprocedural MI as defined by SCAI definition was comparable between the two groups (5.6 vs. 7.1%, *p* = 0.60), while periprocedural myocardial infarction defined by WHO definition is significantly lower in the JBT group than the JWT group (7.0% vs. 14.9%, *p* = 0.03). Periprocedural MI defined by ARC-2 definition was lower in patients in the JBT strategy group (5.6 vs. 11.3%, *p* = 0.08) than that in the JWT group. Nevertheless, no significant differences were detected. Similar trends were also found in the ATS population (Table 5).

**TABLE 5:** Periprocedural myocardial infarction based on different definitions.

	Intention-to-treat population				As-treated population			
	JBT group (*N* = 143)	JWT group (*N* = 141)	Difference (95% CI)*	*P*-value	JBT group (*N* = 140)	JWT group (*N* = 138)	Difference (95% CI)*	*P*-value
Periprocedural MI (SCAI)	5.6% (8)	7.1% (10)	–1.5 (–7.2, 4.2)	0.60	5.7% (8)	7.2% (10)	–1.5 (–7.3, 4.3)	0.60
Periprocedural MI (WHO)	7.0% (10)	14.9% (21)	–7.9 (–15.1, –0.7)	0.02	7.1% (10)	15.2% (21)	–8.1 (–15.4, –0.7)	0.02
Periprocedural MI (ARC-2)	5.6% (8)	11.3% (16)	–5.8 (–12.2, 0.7)	0.08	5.7% (8)	11.6% (16)	–5.9 (–12.5, 0.7)	0.08

Values are n (%). Abbreviation as in Table 1. *The value is the difference between the intentional strategy group and the conventional strategy group.

### One-Year Clinical Outcomes

**FIGURE 3:**
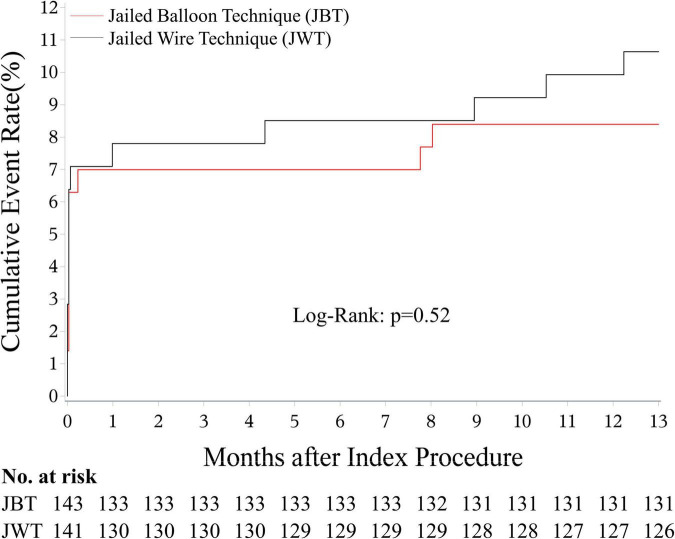
MACE-free survival rate at 1 year. The rate was 91.6% in the JBT group, and it was 89.4% in the JWT group (*p* = 0.52). MACE: major adverse cardiac event; JBT: jailed balloon technique; JWT: jailed wire technique.

Cardiac death (0.7 vs. 0.7%, *p* = NS) was comparable between the two groups. Although no significant differences were detected, the JBT group showed a trend of a lower rate in MI (6.3 vs. 7.1%, *p* = 0.79), target lesion revascularization (TLR, 1.4 vs. 2.1%, *p* = 0.68), and MACE a composite of all-cause death (MI, or TLR, 8.4 vs. 10.6%, *p* = 0.52, Figure 3) than the JWT group. Similar results were found in the ATS population (Table 6).

**TABLE 6:** Clinical outcomes at 1 year^†^.

	Intention-to-treat population				As-treated set			
	JBT group (*N* = 143)	JWT group (*N* = 141)	Difference (95% CI)*	*P*-Value	JBT group (*N* = 140)	JWT group (*N* = 138)	Difference (95% CI)*	*P*-value
MACE	8.4% (12)	10.6% (15)	–2.3 (–9.1, 4.6)	0.52	9.3% (13)	10.1% (14)	–0.9 (–7.8, 6.1)	0.81
All-cause death	0.7% (1)	0.7% (1)	–0.01 (–1.96, 1.94)	1.00	1.4% (2)	0% (0)	1.4 (–0.5, 3.4)	0.50
Cardiac death	0.7% (1)	0.7% (1)	–0.01 (–1.96, 1.94)	1.00	1.4% (2)	0% (0)	1.4 (–0.5, 3.4)	0.50
Myocardial Infarction	6.3% (9)	7.1% (10)	–0.8 (–6.6, 5.0)	0.80	6.4% (9)	7.2% (10)	–0.8 (–6.8, 5.1)	0.79
Periprocedural MI	5.6% (8)	7.1% (10)	–1.5 (–7.2, 4.2)	0.60	5.7% (8)	7.2% (10)	–1.5 (–7.3, 4.3)	0.60
Any revascularization	3.5% (5)	5.0% (7)	–0.1.5 (–6.2, 3.2)	0.54	3.6% (5)	5.1% (7)	–1.5 (–6.3, 3.3)	0.54
TVR	2.1% (3)	2.8% (4)	–0.7 (–4.4, 2.9)	0.72	2.1% (3)	2.9% (4)	–0.8 (–4.4, 2.9)	0.72
TLR	1.4% (2)	2.1% (3)	–0.7 (–3.8, 2.3)	0.68	1.4% (2)	2.2% (3)	–0.8 (–3.9, 2.4)	0.68
Definite/probable stent thrombosis	0.7% (1)	1.4% (2)	–0.7 (–3.1, 1.7)	0.62	0.7% (1)	1.4% (2)	–0.7 (–3.2, 1.7)	0.62
Definite stent thrombosis	0.7% (1)	1.4% (2)	–0.7 (–3.1, 1.7)	0.62	0.7% (1)	1.4% (2)	–0.7 (–3.2, 1.7)	0.62

Values are% (n). *The value is the difference between intentional strategy group and conventional strategy group.

*^†^One year follow-up includes a window of ± 30 days. MACE was defined as a composite of all-cause death, MI (SCAI definition), or TVR. MACE, major adverse cardiac events; TVR, target vessel revascularization; TLR, target lesion revascularization; other abbreviations as in Table 1.*

## Discussion

Although JBT has been proposed for about one decade, no randomized trials have been performed to compare the efficacy between JBT and JWT. As far as we know, the current study is the first and largest randomized data that compared JBT and JWT in high-risk bifurcation. The major findings of the present study are as follows: (1) JBT was associated with a significantly lower incidence of SB occlusion and (2) although no significant difference in 1-year MACE was detected, patients in the JBT group have a significantly lower incidence of WHO defined periprocedural MI.

### Efforts to Protect the Side Branch

Treatment of a bifurcation lesion remains a challenging problem in interventional cardiology due to the possibility of SB failure and higher rates of complications (13). SB occlusion can lead to clinically significant MI and even death. Branch occlusion may affect the PCI procedure directly: SB occlusion prompts interventionalists to urgently restore the branch vessels. During this unexpected episode, rewiring under the intima and suboptimal stent implantation may occur and result in greater rates of in-hospital complications and long-term MACE (2).

Different SB protection strategies have been advocated to minimize the risk for significant SB compromise. A primary two-stent strategy was one of the treatment options for high-risk bifurcation. However, randomized clinical trials and meta-analyses have shown that the single-stent approach was associated with significantly lower rates of all-cause mortality with similar rates of MACE and stent thrombosis compared with two-stent techniques lesions (14). The advantages of the protection SB and the potential disadvantages that may affect the prognosis put the two-stent technology into a dilemma, limiting its wide use.

Jailed balloon technique is a more active type of provisional bifurcation stenting technique than JWT. JBT is designed to improve SB access if the re-intervention of SB after MV stenting is needed. If SB is occluded, the jailed balloon is inflated to restore SB flow. Burzotta et al. proposed the JBT technique and showed that JBT was successful in all 20 patients (6). Singh et al. reported their experiences with JBT in 102 lesions, which showed a very low incidence of SB occlusion of all bifurcation lesions, and patients who underwent JBT have a relatively low incidence of cardiovascular events during follow-up (9). Studies also reported that the modified JBT technique, in which the jailed balloon is simultaneously inflated when the MV stent is deployed, is safe and effective in preserving SB patency (15, 16). However, all these studies were retrospective analyses and no randomized trials have been performed regarding JBT.

### Efficacy and Safety of Jailed Balloon Technique

Previous studies have shown the high efficacy of JBT in protecting SB. The rate of SB occlusion in bifurcation that underwent JBT range from 0% (0/20) (6) to 1% (1/101) (9). In the present study, due to the fact that the included bifurcations are of high risk in SB occlusion and the different definition of SB occlusion (any decrease in TIMI flow grade or absence of flow in SB immediately after full apposition of the MV stent to the vessel wall is considered as SB occlusion), the incidence of SB occlusion in the JBT group is relatively higher than in the previous studies. If the SB occlusion is defined as the absence of blood flow in the SB, the rate of SB occlusion would be 2.8% (4/143). Considering the present study only included bifurcations with high risk in SB occlusion, the SB occlusion rate (2.8%) may be considered as close to those in previous studies. In the present study, patients in the JBT group had a significantly lower rate of SB occlusion than the JWT group in both ITT and ATS set, demonstrating trustworthy efficacy.

Regarding safety, patients in the JBT group have no jailed balloon-related complications in the present study. Similar to jailed wire entrapment that can occur during JWT, one dreaded complication of JBT is the entrapment of the jailed balloon. Although this complication is limited in case reports of severe calcified lesions (17) and was successfully managed by repeated balloon inflation and deflation, the inability to retrieve a balloon may be catastrophic. Therefore, the JBT technique needs to be used cautiously in severe calcified lesions. Another concern of the interventionalists with the JBT is the possibility of stent deformation, which can be managed by using the POT technique. This is why we recommend using the POT technique after the removal of the confinement balloon.

### Jailed Balloon Technique and 1-Year Clinical Outcomes

Side branch occlusion is a serious complication and can result in greater rates of in-hospital complications and long-term MACE. In the present study, JWT reduced the incidence of SB occlusion as well as the WHO defined periprocedural MI. Previous studies have reported that periprocedural MI or even a small increase in cardiac biomarker levels after PCI is associated with a significantly higher risk of late mortality (18–21). However, in the present study, the difference in WHO-defined periprocedural MI did not directly translate into the difference in 1-year MACE. Possible explanations are as follows: (1) the present study is a subgroup analysis and the sample size may not have enough power to detect the potential difference in 1-year MACE; (2) all procedures were performed by experienced interventionalists with an annual volume larger than 200 PCIs, a considerable part of the occluded SB was re-opened and the blood flow was restored. There was no significant difference in SB lesion success (final TIMI flow III was achieved in SB, 93.7% in the JBT group vs. 92.9% in the JWT group, *p* = 0.79). The rich experience of the operators makes patients in both groups have a pronounced prognosis and a relative low incidence of 1-year MACE, which further increases the difficulty of detecting the difference in prognosis.

Interventionalists may be concerned that the jailed balloon of JBT would damage the polymer of the MV stent and cause adverse events. In the present study, no relevant events were proved to be related to the damaged polymer. Perhaps, further studies with intravascular imaging may help to clear this issue.

### Limitations

Despite being the first and largest randomized data to compare JBT and JWT in high-risk bifurcation, the present study has some limitations that should be acknowledged. First, the present study is a subgroup analysis of the CIT-RESOLVE trial with its inherent limitations. The CIT-RESOLVE trial was prematurely terminated because of slow recruitment. Although the *post hoc* calculation of the conditional power confirmed that the sample size of CIT-RESOLVE had enough power (8), the cohort of the present subgroup was not powered for showing a difference in the primary outcome between JBT and JWT groups. Second, a modified JBT technique has been proposed in 2018 and is considered to have less stent deformity than the original JBT (15). The current study was performed from December 2016 to April 2019 and used the JBT technique in its original form. Thus, further studies are warranted. Third, the overall rates of POT were low, and as such could have influenced the results. Lastly, in the current study, the assessment of whether the lesion is successfully treated relied solely on coronary angiography, with no mandatory intravascular imaging or functional evaluation. The use of intravascular imaging and functional evaluation is warranted in further studies.

## Conclusion

In patients with a high risk of SB occlusion (V-RESOLVE score ≥ 12 points), JBT is superior to JWT in reducing SB occlusion. However, no significant differences were detected in a 1-year MACE.

## Data Availability Statement

The original contributions presented in the study are included in the article/Supplementary Material, further inquiries can be directed to the corresponding author/s.

## Ethics Statement

The studies involving human participants were reviewed and approved by the Ethics Committee of the Cardiovascular Institute and Fuwai Hospital. The patients/participants provided their written informed consent to participate in this study.

## Author Contributions

All authors were fully involved in the study and preparation of the manuscript, and contributed significantly to the submitted work, in terms of conception and design of the study, analysis and interpretation of the results and critical review.

## Conflict of Interest

The authors declare that the research was conducted in the absence of any commercial or financial relationships that could be construed as a potential conflict of interest.

## Publisher’s Note

All claims expressed in this article are solely those of the authors and do not necessarily represent those of their affiliated organizations, or those of the publisher, the editors and the reviewers. Any product that may be evaluated in this article, or claim that may be made by its manufacturer, is not guaranteed or endorsed by the publisher.

## Abbreviations

ATS: as-treated set
ITT: intention-to-treat
JBT: jailed balloon technique
JWT: jailed wire technique
MACE: major adverse cardiac events
MI: myocardial infarction
MV: main vessel
PCI: percutaneous coronary intervention
QCA: quantitative coronary angiography
RVD: reference vessel diameter
SB: side branch
TIMI: thrombolysis in myocardial infarction.
