# Supplementary material for: Jailed Balloon Technique Is Superior to Jailed Wire Technique in Reducing the Rate of Side Branch Occlusion: Subgroup Analysis of the Conventional Versus Intentional StraTegy in Patients With High Risk PrEdiction of Side Branch OccLusion in Coronary Bifurcation InterVEntion Trial
Source: Front Cardiovasc Med. 2022 Mar 31;9:814873. doi: 10.3389/fcvm.2022.814873 (PMC9008226; doi:10.3389/fcvm.2022.814873)
Supplement: Supplementary file 1 [file Data_Sheet_1.docx]

**Jailed Balloon Technique is Superior to Jailed Wire Technique in Reducing the Rate of Side Branch Occlusion: Subgroup Analysis of the CIT-RESOLVE Trial**

Dong Zhang^1, 2,3^, MD; Zhiyong Zhao^1, 2,3^, MD; Guofeng Gao^1, 2,3^, MD; Han Xu^1, 2,3^, MD; Hao Wang^1, 2,3^, MD; Shuai Liu^1, 2,3^, MD; Dong Yin^1, 2,3^, MD; Lei Feng^1, 2,3^, MD; Chenggang Zhu^1, 2,3^, MD; Yang Wang^4^, MSc; Yanyan Zhao^4^, MSc; Yuejin Yang^1, 2,3^, MD; Runlin Gao^1, 2,3^, MD; Bo Xu^1, 3, 5^, MBBS; Kefei Dou^1, 2,3^, MD

From the ^1^State Key Laboratory of Cardiovascular Disease, Beijing, China; ^2^Department of Cardiology, Fu Wai Hospital, National Center for Cardiovascular Diseases, ^3^Chinese Academy of Medical Sciences and Peking Union Medical College, Beijing, China; ^4^Medical Research and Biometrics Center, National Center for Cardiovascular Diseases, Beijing, China; ^5^Catheterization Laboratories, Fu Wai Hospital, Beijing, China.

**Address for Correspondence:**

Kefei Dou, MD

State Key Laboratory of Cardiovascular Disease

Department of Cardiology, Fu Wai Hospital

National Center for Cardiovascular Diseases

Chinese Academy of Medical Sciences and Peking Union Medical College

National Clinical Research Center for Cardiovascular Diseases

A 167, Beilishi Road, Xicheng District

Beijing, 100037, China

Tel: +86-10-8839-6590, Fax: +86-10-6831-3012

E-mail: [drdoukefei@126.com](mailto:drdoukefei@126.com)

And

Bo Xu, MBBS

Catheterization Laboratories, Fu Wai Hospital

National Center for Cardiovascular Diseases

Chinese Academy of Medical Sciences and Peking Union Medical College

National Clinical Research Center for Cardiovascular Diseases

A 167, Beilishi Road, Xicheng District

Beijing, 100037, China

Tel: +86-10-8832-2562, Fax: +86-10-6831-3012

E-mail: [bxu@citmd.com](mailto:bxu@citmd.com)

Table S1. Baseline Patient and Lesion Characteristics (As Treated Set)

|  | **Jailed Balloon Group**  **(N=140)** | **Jailed Wire Group**  **(N=138)** | **p Value** |
| --- | --- | --- | --- |
| Age, years | 60.4 ± 8.8 | 61.3 ± 10.1 | 0.43 |
| Male | 73.6% (103) | 68.8% (95) | 0.38 |
| Body mass index | 27.5 ± 27.3 (135^*^) | 25.4 ± 6.0 (134^*^) | 0.39 |
| Diabetes mellitus | 29.3% (41) | 28.3% (39) | 0.85 |
| Hypertension | 57.9% (81) | 65.9% (91) | 0.16 |
| Hyperlipidemia | 40.0% (56) | 38.4% (53) | 0.79 |
| Current smoker | 45.7% (64) | 42.8% (59) | 0.62 |
| Previous MI | 21.4% (30) | 24.6% (34) | 0.53 |
| Previous PCI | 13.6% (19) | 19.6% (27) | 0.18 |
| Previous CABG | 0.7% (1) | 0.7% (1) | 1.0 |
| Family history of CAD | 8.6% (12) | 12.3% (17) | 0.31 |
| Peripheral arterial disease | 8.6% (12) | 7.2% (10) | 0.68 |
| Unstable angina | 67.1% (94) | 54.3% (75) | 0.03 |
| Left ventricular ejection fraction | 63.1 ± 8.4 (131^*^) | 61.2 ± 9.5 (125^*^) | 0.09 |
| Multivessel disease | 53.6% (75) | 52.2% (72) | 0.82 |
| Target lesion location |  |  |  |
| Left anterior descending/diagonal | 87.9% (123) | 81.2% (112) | 0.12 |
| Left circumflex/obtuse marginal | 10.7% (15) | 14.5% (20) | 0.34 |
| Right coronary artery bifurcation | 1.4% (2) | 4.3% (6) | 0.17 |
| Medina Type |  |  |  |
| 1,0,0 | 2.1% (3) | 2.2% (3) | 1.0 |
| 0,1,0 | 1.4% (2) | 1.4% (2) | 1.0 |
| 1,1,0 | 7.9% (11) | 4.3% (6) | 0.22 |
| 1,1,1 | 61.4% (86) | 63.8% (88) | 0.69 |
| 0,0,1 | 0% (0) | 0% (0) | - |
| 1,0,1 | 10.7% (15) | 10.9% (15) | 0.97 |
| 0,1,1 | 16.4% (23) | 17.4% (24) | 0.83 |
| ACC/AHA class B2/C lesions | 95.0% (133) | 96.4% (133) | 0.57 |
| Baseline SYNTAX score | 17.3 ± 7.5 (138^*^) | 18.5 ± 8.0 (135^*^) | 0.19 |
| MV qualitative analysis |  |  |  |
| Baseline TIMI flow |  |  | 0.18 |
| 0 | 3.6% (5) | 5.8% (8) |  |
| I | 4.3% (6) | 8.0% (11) |  |
| II | 3.6% (5) | 7.2% (10) |  |
| III | 88.6% (124) | 79.0% (109) |  |
| In-stent restenosis | 0.7% (1) | 1.4% (2) | 0.62 |
| Total occlusion | 2.9% (4) | 5.8% (8) | 0.22 |
| Moderate or heavy calcification | 7.9% (11) | 9.4% (13) | 0.64 |
| Severely tortuous or angulated lesion | 20.7% (29) | 20.3% (28) | 0.93 |
| Thrombus containing | 1.4% (2) | 0 (0) | 0.50 |
| Plaque located at the same side of SB | 92.1% (129) | 91.3% (126) | 0.80 |
| Irregular plaque | 56.4% (79) | 59.4% (82) | 0.61 |
| SB qualitative analysis |  |  |  |
| Baseline TIMI flow |  |  | 0.90 |
| 0 | 0% (0) | 0.7% (1) |  |
| I | 3.6% (5) | 2.9% (4) |  |
| II | 5.0% (7) | 5.8% (8) |  |
| III | 91.4% (128) | 90.6% (125) |  |
| In-stent restenosis | 0% (0) | 0% (0) | - |
| Total occlusion | 0% (0) | 0.7% (1) | 0.50 |
| Moderate or heavy calcification | 0 (0) | 2.2% (3) | 0.12 |
| Severely tortuous or angulated lesion | 12.1% (17) | 13.8% (19) | 0.69 |
| Thrombus containing | 0% (0) | 0% (0) | - |
| Irregular plaque | 42.9% (60) | 28.3% (39) | 0.01 |
| V-RESOLVE score (site) | 15.5 ± 3.2 (140^*^) | 15.6 ± 3.1 (138^*^) | 0.85 |
| V-RESOLVE score (core lab) | 17.3 ± 3.4 (140^*^) | 17.0 ± 4.1 (138^*^) | 0.60 |

Values are mean± SD or % (n). ^*^Number of patients for whom continuous variables were calculated.

MI = myocardial infarction; PCI = percutaneous coronary intervention; CABG = coronary artery bypass grafting; CAD = coronary artery disease; ACC = American College of Cardiology; AHA = American Heart Association; SYNTAX = Synergy Between PCI With TAXUS and Cardiac Surgery; MV = main vessel; TIMI = Thrombolysis In Myocardial Infarction; V-RESOLVE = Visual estimation for Risk prEdiction of Side branch OccLusion in coronary bifurcation intervention; other abbreviations as in Table 1.

Table S2. Procedural Characteristics and Results (As Treated Set)

|  | **Jailed Balloon Group**  **(N=140)** | **Jailed Wire Group**  **(N=138)** | **Difference**  **(95% CI)**^*^ | **p Value** |
| --- | --- | --- | --- | --- |
| Transradial approach | 96.4% (135) | 95.6% (131) | 0.81 (-3.8, 5.4) | 0.73 |
| Nitroglycerin use | 33.6% (47) | 27.5% (38) | 6.0 (-4.8, 16.8) | 0.27 |
| Dopamine use | 0.7% (1) | 0% (0) | 0.7 (-0.7, 2.1) | 1.00 |
| MV |  |  |  |  |
| Balloon pre-dilation | 100% (140) | 98.6% (136) | 1.5 (-0.5, 3.4) | 0.25 |
| Maximal diameter of pre-dilation balloon, mm | 2.2 ± 1.3 | 2.50 ± 1.9 | -0.3 (-0.6, 0.0) | 0.06 |
| Maximal inflation pressure with pre-dilation balloon, atm | 12.0 ± 2.5 | 11.9 ± 2.7 | 0.06 (-0.57, 0.69) | 0.85 |
| Dissection before MV stenting | 2.1% (3) | 4.3% (6) | -2.2 (-6.4, 2.0) | 0.33 |
| Number of stents in MV | 1.3 ± 0.5 | 1.3 ± 0.5 | 0.04 (-0.07, 0.15) | 0.48 |
| Stent diameter in MV, mm | 3.0 ± 0.4 | 3.0 ± 0.3 | 0.06 (-0.03, 0.14) | 0.18 |
| Total stent length in MV, mm | 26.8 ± 7.9 | 27.5 ± 8.7 | -0.6 (-2.6, 1.4) | 0.54 |
| Lesion success | 99.3% (139) | 99.3% (137) | 0.01 (-1.98, 2.00) | 1.00 |
| SB |  |  |  |  |
| Balloon pre-dilation | 37.9% (53) | 34.1% (47) | 3.8 (-7.5, 15.1) | 0.51 |
| SB stenting |  |  |  |  |
| Elective 2-stent strategy | 0 (0) | 0 (0) | - | - |
| Provisional SB stenting | 0.7% (1) | 2.2% (3) | -1.4 (-4.3, 1.3) | 0.37 |
| Number of stents in SB | 0 | 0.04 ± 0.22 | -0.04 (-0.07, 0) | 0.37 |
| Treatment after MV stent deployed | 45.7% (64) | 33.3% (46) | 12.4 (0.1, 23.8) | 0.04 |
| Final kissing balloon inflation | 18.6% (26) | 18.8% (26) | -0.3 (-9.4, 8.9) | 0.95 |
| Lesion treated with POT | 31.4% (43) | 39.9% (41) | 1.5 (-9.5, 12.4) | 0.79 |
| Lesion treated with re-POT | 3.6% (5) | 1.5% (2) | 2.2 (-1.5, 5.9) | 0.44 |
| Lesion success | 93.6% (131) | 93.5% (129) | 0.1 (-5.7, 5.9) | 0.97 |

Values are mean± SD or % (n). ^*^The value is the difference between intentional strategy group and conventional strategy group.

CI = confidence interval; RVD = reference vessel diameter; other abbreviations as in Table S1.

Table S3. Quantitative Coronary Angiographic Results

|  | **Intention-to-Treat Population** | | | | **As Treated Set** | | | |
| --- | --- | --- | --- | --- | --- | --- | --- | --- |
|  | **JBT Group** | **JWT Group** | **Difference**  **(95% CI)^*^** | **p Value** | **JBT Group** | **JWT Group** | **Difference**  **(95% CI)^*^** | **p Value** |
| Pre-procedure QCA | N=130^†^ | N=135^†^ |  |  | N=127^†^ | N=132^†^ |  |  |
| Proximal MV |  |  |  |  |  |  |  |  |
| RVD, mm | 2.97 ± 0.53 | 2.88 ± 0.50 | 0.08 (-0.04, 0.21) | 0.18 | 2.93 ± 0.53 | 2.92 ± 0.50 | 0.01 (-0.11, 0.14) | 0.85 |
| Diameter stenosis, % | 46.57 ± 23.76 | 49.50 ± 25.14 | -2.93 (-8.86, 2.99) | 0.33 | 46.08 ± 24.31 | 49.77 ± 24.98 | -3.69 (-9.72, 2.35) | 0.23 |
| Lesion length, mm | 9.06 ± 7.35 | 8.87 ± 6.67 | 0.19 (-1.51, 1.88) | 0.83 | 8.99 ± 7.45 | 8.98 ± 6.71 | 0 (-1.73, 1.74) | 1.0 |
| Distal MV |  |  |  |  |  |  |  |  |
| RVD, mm | 2.41 ± 0.54 | 2.24 ± 0.48 | 0.17 (0.05, 0.3) | 0.005 | 2.39 ± 0.54 | 2.26 ± 0.49 | 0.13 (0.01, 0.26) | 0.04 |
| Diameter stenosis, % | 50.05 ± 19.07 | 47.90 ± 20.53 | 2.15 (-2.66, 6.95) | 0.38 | 50.62 ± 18.36 | 47.84 ± 21.20 | 2.79 (-2.08, 7.66) | 0.26 |
| Lesion length, mm | 6.95 ± 5.47 | 7.15 ± 5.66 | -0.20 (-1.55, 1.15) | 0.77 | 7.18 ± 5.79 | 7.00 ± 5.46 | 0.18 (-1.2, 1.56) | 0.80 |
| SB |  |  |  |  |  |  |  |  |
| RVD, mm | 1.83 ± 0.43 | 1.77 ± 0.38 | 0.06 (-0.04, 0.15) | 0.27 | 1.80 ± 0.43 | 1.79 ± 0.37 | 0.01 (-0.09, 0.11) | 0.89 |
| Diameter stenosis, % | 42.00 ± 18.22 | 41.28 ± 19.39 | 0.72 (-3.84, 5.29) | 0.76 | 41.54 ± 18.35 | 41.17 ± 19.47 | 0.37 (-4.27, 5.02) | 0.87 |
| Lesion length, mm | 5.57 ± 3.60 | 4.95 ± 3.63 | 0.61 (-0.26, 1.49) | 0.17 | 5.53 ± 3.65 | 4.88 ± 3.61 | 0.66 (-0.23, 1.55) | 0.15 |
| During-procedure QCA | N=118^†^ | N=133^†^ |  |  | N=115^†^ | N=131^†^ |  |  |
| Diameter stenosis of SB before MV stenting (%) | 41.74 ± 19.55 | 39.50 ± 18.26 | 2.24 (-2.46, 6.94) | 0.35 | 41.77 ± 19.51 | 39.32 ± 18.35 | 2.45 (-2.31, 7.21) | 0.31 |
| Diameter stenosis of MV before MV stenting (%) | 51.77 ± 13.54 | 52.83 ± 13.78 | -1.05 (-4.46, 2.35) | 0.54 | 51.40 ± 13.60 | 53.03 ± 13.79 | -1.63 (-5.08, 1.82) | 0.35 |
| Post-procedure QCA | N=132^†^ | N=135^†^ |  |  | N=129^†^ | N=132^†^ |  |  |
| Diameter stenosis in proximal MV, % | 12.25 ± 8.44 | 12.68 ± 8.48 | -0.43 (-2.47, 1.61) | 0.68 | 12.44 ± 8.49 | 12.49 ± 8.47 | -0.04 (-2.11, 2.02) | 0.97 |
| Diameter stenosis in distal MV, % | 14.92 ± 9.05 | 14.18 ± 7.24 | 0.74 (-1.23, 2.72) | 0.46 | 14.70 ± 9.15 | 14.24 ± 7.21 | 0.46 (-1.54, 2.47) | 0.65 |
| Diameter stenosis in SB, % | 37.77 ± 21.92 | 40.23 ± 20.46 | -2.46 (-7.57, 2.65) | 0.34 | 38.45 ± 21.51 | 39.96 ± 20.79 | -1.51 (-6.66, 3.65) | 0.57 |
| MLD in proximal MV, mm | 2.64 ± 0.46 | 2.59 ± 0.40 | 0.05 (-0.05, 0.15) | 0.35 | 2.62 ± 0.46 | 2.61 ± 0.39 | 0.01 (-0.09, 0.11) | 0.85 |
| MLD in distal MV, mm | 2.07 ± 0.40 | 2.07 ± 0.34 | 0.00 (-0.09, 0.09) | 0.98 | 2.07 ± 0.39 | 2.07 ± 0.34 | 0.00 (-0.09, 0.09) | 0.98 |
| MLD in SB, mm | 1.01 ± 0.40 | 1.042 ± 0.43 | -0.03 (-0.13, 0.07) | 0.58 | 0.98 ± 0.36 | 1.05 ± 0.43 | -0.07 (-0.17, 0.02) | 0.14 |

Values are mean± SD or % (n). ^*^The value is the difference between intentional strategy group and conventional strategy group. ^†^Number of patients for whom angiographic images were qualified for quantitative coronary angiographic analysis.

MLD = minimal lumen diameter; other abbreviations as in Tables S1 and S2
